# An enzyme free simultaneous detection of γ-amino-butyric acid and testosterone based on copper oxide nanoparticles†

**DOI:** 10.1039/d1ra02709c

**Published:** 2021-06-11

**Authors:** Mohammad Musarraf Hussain, Abdullah M. Asiri, Jamal Uddin, Mohammed M. Rahman

**Affiliations:** Department of Chemistry, Faculty of Science, King Abdulaziz University P. O. Box 80203 Jeddah 21589 Saudi Arabia m.musarraf.hussain@gmail.com mmrahman@kau.edu.sa mmrahmanh@gmail.com; Center of Excellence for Advanced Materials Research (CEAMR), King Abdulaziz University P. O. Box 80203 Jeddah 21589 Saudi Arabia; Department of Pharmacy, Faculty of Life and Earth Sciences, Jagannath University Dhaka-1100 Bangladesh mmhussain@pharm.jnu.c.bd; Center for Nanotechnology, Department of Natural Sciences, Coppin State University Baltimore MD 21216 USA

## Abstract

Herein, an easy wet-chemical process was used in basic medium with low temperature to prepare low-dimensional copper oxide nanoparticles (CuO NPs). A variety of optical and structural techniques such as UV-visible, FT-IR, XRD, FESEM, XEDS, and XPS were used to characterize the synthesized CuO NPs in detail. Two sensitive and selective sensor probes for γ-amino-butyric acid (GABA) and testosterone (TST) were achieved after modification; a thin layer of NPs on a flat glassy carbon electrode (GCE). Sensor analytical parameters such as sensitivity (SNT), linear dynamic range (LDR), limit of detection (LOD), limit of quantification (LOQ), robustness, and interference effects, were evaluated for the proposed sensor (GCE/CuO NPs) for GABA and TST, based on a dependable current–voltage technique. Calibration curves were found to be linear (*R*^2^ = 0.9963 and 0.9095) over a broad concentration range of GABA and TST (100.0 pM to 100.0 mM and 10.0 pM to 10.0 mM, respectively). Sensor parameters – SNT (316.46 and 2848.10 pA μM^−1^ cm^−2^), LDR (100.0 nM to 10.0 mM and 10.0 pM to 1.0 mM), LOD (≈11.70 and 96.67 pM), and LOQ (39.0 and 322.2 pM) – for GABA and TST were calculated from the calibration plot successively. Preparation of CuO NPs using the wet-chemical technique is a good approach for perspective expansion of NPs-based sensors for the enzyme-free detection of biomolecules. Our sensor probe (GCE/CuO NPs) is applied for the cautious recognition of GABA and TST in real biological samples –human, mouse, and rabbit serum – and achieved good and acceptable results.

## Abstract 2

An easy wet-chemical process was used to prepare copper oxide nanoparticles which were modified and used as sensor probes for γ-amino-butyric acid and testosterone.

## Graphical abstract

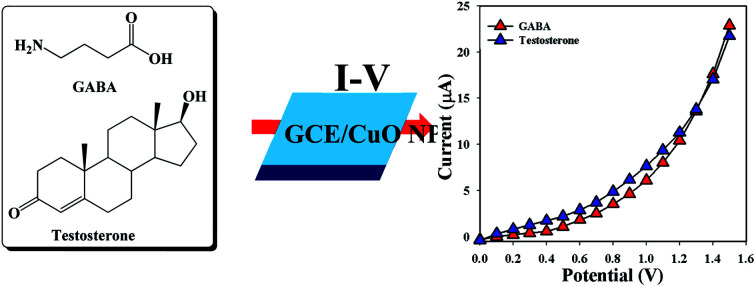

## 1. Introduction

GABA (γ-amino-butyric acid) is a four carbon, non protein, organic amino acid which is a significant component in the free amino acid collection and is found in eukaryotes and prokaryotes. It is commonly dispersed in the brain and is reported to make-up 30–40% of entire synapses. GABA is mainly present in the amygdala, basal ganglia, cerebellum, cortex, hypothalamus, hippocampus, spinal cord, and medulla. It possesses several biological functions, for example diuretic and antihypertensive effects, lowering blood pressure, development of brain activities, and tranquillizing functions. A deep sleep may be achieved by taking GABA as a supplement before bed time.^1,2^ It is a key neurotransmitter in the brain, and is associated with inhibition. Psychiatric and neurological disorders along with convulsions, epilepsy, Huntington’s disease, Parkinsonism, and seizures can result from GABA concentration changes.^3^

Testosterone (TST) is a major androgenic hormone that controls numerous physiological procedures, for example bone, muscle protein and lipid metabolism, cardiac contractile activities, human performance, and sexual characteristics.^4^ TST deficiency can result in cardiovascular and coronary artery diseases and deficient left ventricular activity.^5^

Traditional techniques have been developed for the measurement of GABA concentration *in vitro* such as HPLC-electrochemical, HPLC-fluorescence, capillary electrophoresis-laser induced fluorescence, immunoassay, HPLC-MS, and UHPLC. Monitoring of TST levels in biological fluids is significant for biochemical, clinical, and sports endocrinology exploration; and immunoassay techniques such as capillary electrophoresis, electrochemistry, electrochemical techniques including luminescence and surface plasmon resonance, have been reported for the recognition of TST. However, these procedures are expensive and require time consuming sample preparation as activation, amplification, derivation, and selection are required. So, designing a new sensing method with good detection and reliability in comparison with sophisticated laboratory instruments is a significant development in this arena.^6,7^ The aim of this research work is to design a selective biochemical sensor probe for the simultaneous detection of γ-amino-butyric acid and testosterone based on copper oxide nanoparticles in enzyme-free conditions.

## 2. Experimental section

### 2.1 Materials and methods

Copper oxide (CuO), γ-amino-butyric acid, ascorbic acid, cholesterol, dopamine, l-glutamic acid, l-leucine, l-tyrosine, testosterone, and uric acid, EtOH, Nafion, and NaOH were purchased as analytical grade chemicals from Sigma-Aldrich (KSA) and used without further purification. UV-visible and FT-IR spectra of the prepared CuO NPs were recorded using a Thermo scientific 300k UV-visible spectrophotometer and NICOLET iS50 FT-IR spectrometer (Madison, USA). XRD testing was carried out in an ambient environment for the determination of the crystalline character of CuO NPs. FESEM (JEOL, JSM-7600F, Japan) equipped with XEDS was used to examine the electrochemical criteria such as arrangement, elemental analysis, morphology, and particle size of the CuO NPs. Binding energies between the elements (Cu and O) were determined based on XPS [Thermo scientific A1 K-α1 1066 spectrometer with an excitation radiation resource (beam spot size = 300.0 μm, pressure = 10^−8^ torr, and pass energy = 200.0 eV)]. JEM-1400FLASH (TEM) was used to analyse the sample for surface analysis. Current–voltage (*I*–*V*) conducting tests were achieved with an electrometer (Keithley, USA) having a selective point regarding measurement of the sensitive and selective bio-molecules. Informed consent was obtained from the volunteers who gave human serum samples, and the mouse and rabbit were purchased from the local market in Jeddah, Saudi Arabia and we collected the serum samples from them. The procedures were carried out in accordance with relevant laws including WHO's standard operational guidance for ethics review of health research with human participants, 2011 and local guidelines (ethics guidelines approved by King Abdulaziz University Medical Centre, Saudi Arabia).

### 2.2 Preparation of CuO NPs

**Scheme 1:**
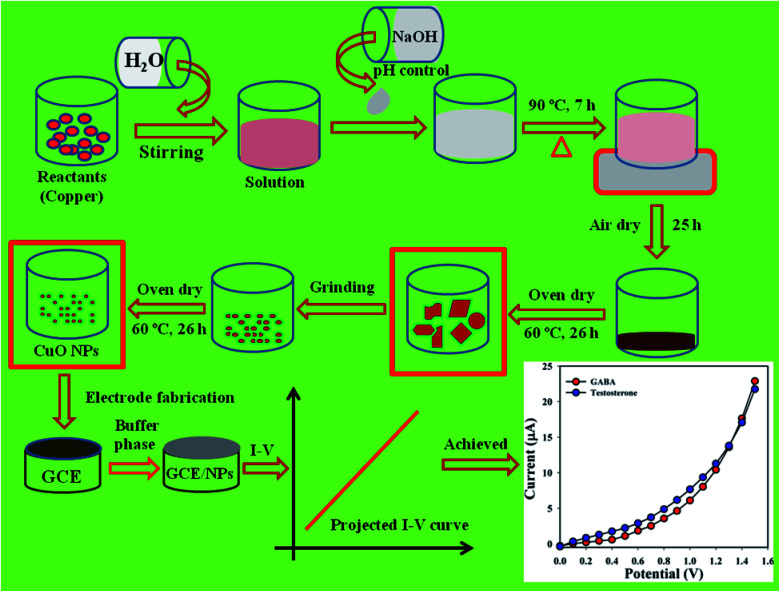
Preparation of CuO NPs by wet-chemical process.

In this analysis, CuSO_4_·5H_2_O and NaOH were used as reactants in the preparation of CuO NPs using a simple wet-chemical procedure.^8,9^ Based on this process, a solution of CuSO_4_·5H_2_O was prepared with distilled water (100.0 mL) and the pH of the consequent solution was altered to be above 10.0 upon addition of NaOH. Then, following 7.0 h continuous stirring using a hot plate (90.0 °C), the conical flask (250.0 mL) was washed systematically with distilled water and acetone and kept for drying in open air at RT (25.0 h). According to Scheme 1, the prepared slurry of CuO NPs was dried in the oven (60.0 °C, 26.0 h), ground into powder, and again dried in the oven (60.0 °C, 26.0 h) prior to use in the electrochemical experiments. A probable mechanism of reactions in the preparation of CuO NPs is as follows in (i)–(iv).^10,11^

i NaOH → Na^+^ + OH^−^

ii CuSO_4_ → Cu^2+^ + SO_4_^2−^

iii Na^+^ + 2OH^−^ + Cu^2+^ + SO_4_^−^ → Cu(OH)_2(aq)_ + NaSO_4(s)_

iv Cu(OH)_2(aq)_ → CuO_(s)_↓ + H_2_O

### 2.3 Electrode modification with CuO NPs

Based on the method introduced by Hussain and co-workers, the requisite number of glassy carbon electrodes (GCEs) were prepared and prepared by a wet-depositing technique.^12–14^ Accordingly, a sequence of phosphate buffer phases from light acidic to basic concentrations (pH = 5.7, 6.5, 7.0, 7.5, and 8.0) were prepared with distilled water, Na_2_HPO_4,_ and NaH_2_PO_4_. GCE is washed systematically with distilled water and acetone, and placed in air (1.0 h). The large exterior of the dried electrodes was covered with the slurry which was prepared from CuO NPs mixed with EtOH, and then kept in open air again to dry (1.0 h). Conducting matrix (Nafion) was added with the flat GCE drop wise and placed again in air (1.0 h) to harmonize the coating growth. Platinum wire and modified GCE were used as counter and working electrode, respectively, for recording the electrical responses to detect the biomolecules.

## 3. Results and discussion

### 3.1 Examination of optical characteristics

**Fig. 1:**
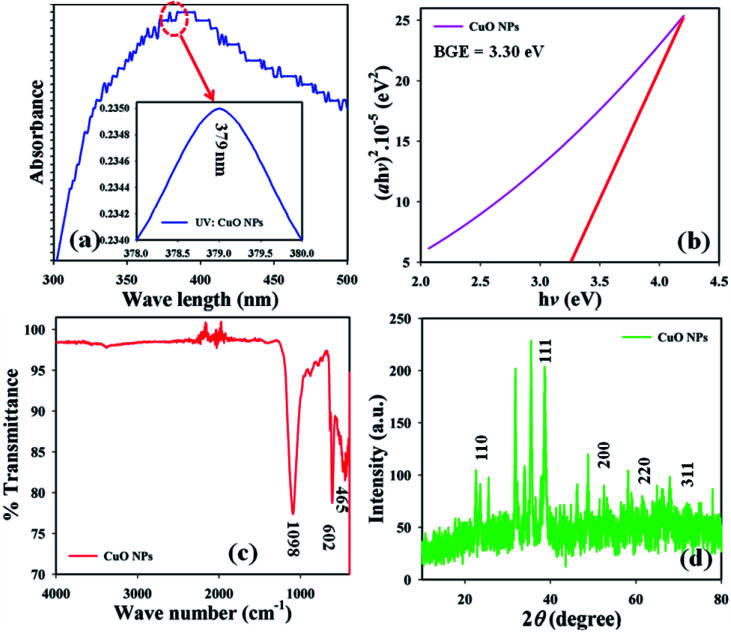
(a) UV-visible, (b) band gap energy plot, (c) FT-IR spectrum, and (d) XRD pattern of CuO NPs.

Optical criteria provide unique information concerning the examination of the photocatalytic features of the prepared CuO NPs. A comprehensive absorption UV-visible curve of the CuO NPs was recorded in the range 200.0–600.0 nm. The UV-visible curve of CuO NPs was plotted in the range 300–500 nm to find out the maximum absorbance (*λ*_max_), which was found to be 379.0 nm. An extended UV-visible curve was plotted between 378–380 nm (Fig. 1a). The theoretical band-gap energy of the CuO NPs (3.27 eV) was achieved based on Tauc’s equation ((v)). Subsequently ℏ*v vs.* (*α*ℏ*v*)^2^ was plotted and then extended to the *x*-axis (eqn (vi)–(viii)) for the determination of the real band-gap energy of the CuO NPs (BGE = 3.30 eV) (Fig. 1b). Here, *A* = constant associated with the valuable mass of the electrons, ℏ = Plank’s constant, *v* = frequency, *α* = absorption coefficient, and *r* = 0.5 (direct transition).^15,16^v
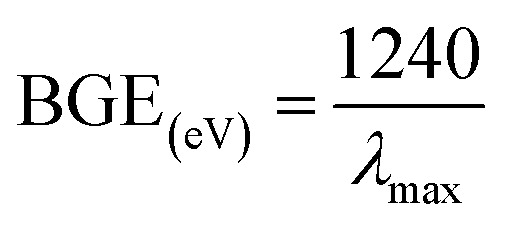

vi ℏ*v* = *A*(*α*ℏ*v*)^1/*r*^

vii ℏ*v* = *A*(*α*ℏ*v*)^2^

viii ℏ*v* ∞ (*α*ℏ*v*)^2^

FT-IR spectrum was conducted at 4000–400 cm^−1^ in order to identify the functional characteristics of the CuO NPs at ambient conditions, using molecular and atomic data. The observed peaks at 1098 and 602 cm^−1^ show the presence of Cu=O. The peak at 465 cm^−1^ shows the presence of Cu=O in the NPs (Fig. 1c). The peaks at 465 cm^−1^ and 602 cm^−1^ were noted as they show the existence of metal oxide bonds (Cu=O), which indicates the prepared CuO NPs.

XRD experimentation was performed (2*θ* = 10–80°) to identify the crystallinity in the synthesized CuO NPs. Strong peaks for 2*θ* were found at 110, 111, 200, 220, and 311 based on the JCPDS 5-0667 file (Fig. 1d). According to the XRD, a high quantity of crystalline copper oxide is present in the synthesized NPs. The standard crystallite dimension (311.28 nm) of the CuO NPs was achieved using the Scherer equation (ix), in this equation, Dp = standard crystallite dimension in nm, *λ* = X-ray wavelength in Å, *θ* = Bragg angle (°), and *β* = line augmentation in radians.^17,18^ix
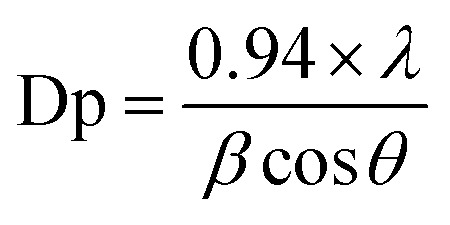


### 3.2 Morphological and elemental characteristics assessment

**Fig. 2:**
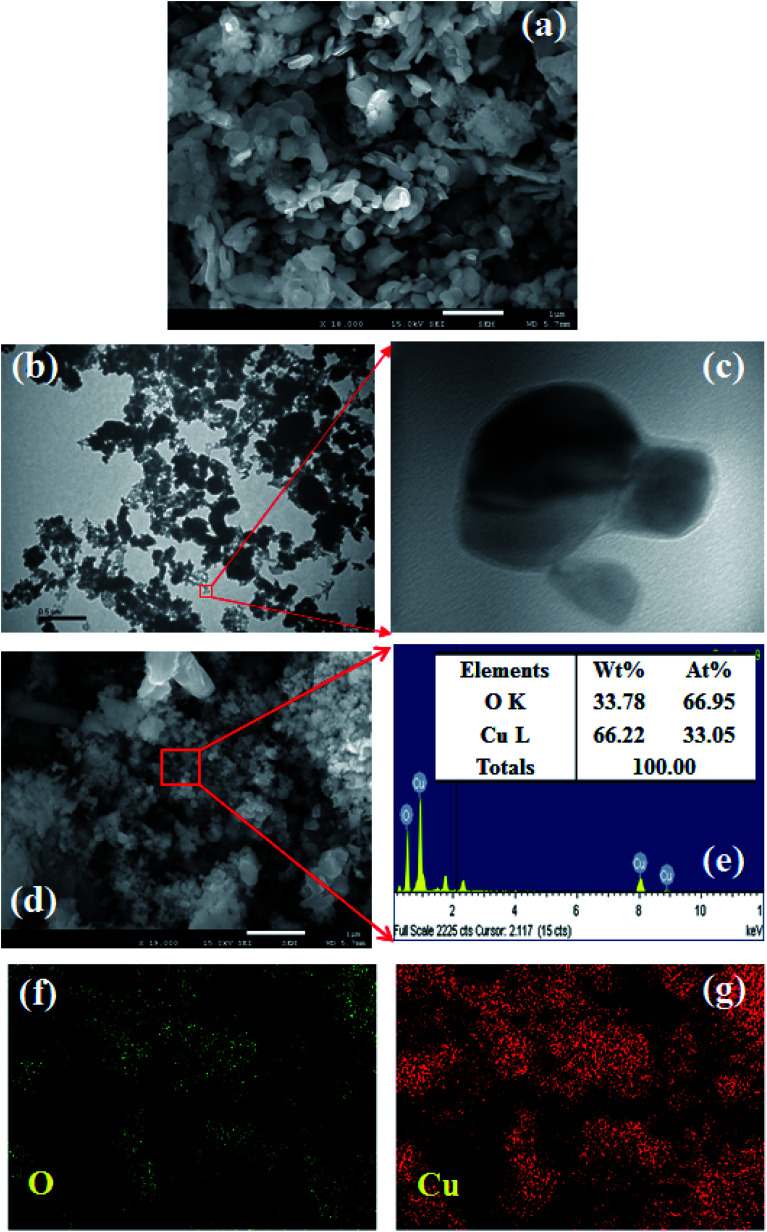
Analyses of CuO NPs, (a) FESEM, (b and c) low to high magnified TEM images, (d and e) elemental analysis (inset: elemental compositions of O and Cu), and (f and g) elemental mapping of O and Cu.

Morphological characteristics of the prepared CuO NPs were examined using FESEM connected with XEDS. A clear FESEM image of the aggregated CuO NPs was recorded and is presented in Fig. 2a. According to the FESEM analysis, the average diameter of CuO NPs is 41.7 nm, in the range 30.0 to 45.0 nm. TEM images were also recorded and provided low to high magnified images of CuO NPs as presented in Fig. 2b and c. The average size calculated from the TEM image is 38.9 nm. According to the XEDS elemental analysis, copper (Cu) and oxygen (O) exist in the prepared CuO NPs (Fig. 2d). The elemental spectrum and composition of each element in the CuO NPs was found in weight percent [Cu L (66.22) and O K (33.78)] and atomic percent [Cu L (66.95) and O K (33.05)], which are presented in Fig. 2e (inset: elemental compositions). No additional peaks were found regarding any impurities in CuO, which suggests the spherical particle was composed of copper and oxygen only. Besides that, the elemental mapping is also analyzed and presented in Fig. 2f and g. The O and Cu elements are dispersed and exist in the whole surface of CuO NPs.

### 3.3 Assessment of binding energies using XPS experiments

**Fig. 3:**
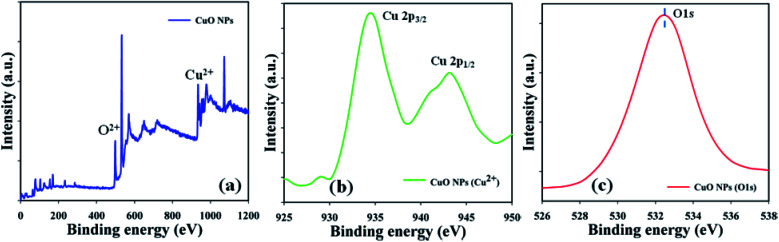
Binding energy examination of CuO NPs (a) full spectrum, (b) Cu^2+^, and (c) O 1s.

The material nature of the components in the prepared CuO NPs was assessed using a quantitative spectroscopic technique (XPS). Electron counting including the kinetic energy of an object may be estimated during XPS inspection, where an X-ray beam can be aggravated in a nanoparticle. Copper and oxygen were found to be present in the prepared CuO NPs, based on the XPS experiment results (Fig. 3a). Spin orbit copper and oxygen were found in the key peaks such as Cu 2p_3/2_ (935.0 eV), Cu 2p_1/2_ (944.0 eV), and O 1s (532.0 eV), that signified that copper (Cu^2+^) and oxygen (O^2−^) were present in the prepared CuO NPs (Fig. 3b and c).

### 3.4. Application

#### 3.4.1 Detection of GABA and TST based on CuO NPs

**Scheme 2:**
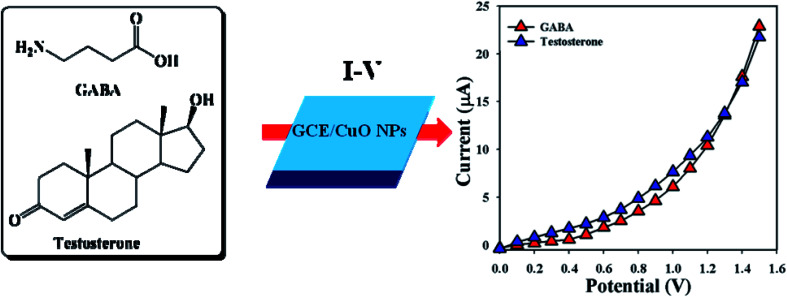
Proposed mechanism regarding detection of GABA and testosterone using CuO NPs.

Designing a sensor according to NPs modified with GCE is a starting point for researchers designing biological sensors. CuO NPs modified electrodes were examined in buffer phase to assess their ability to detect biomolecules such as γ-amino-butyric acid and testosterone. Electrical responses of the proposed sensor (GCE/CuO NPs) were vigorously changed from the perspective of adsorption of GABA and TST throughout the *I*–*V* progression. A proposed mechanism of electrochemical detection of GABA and TST using the *I*–*V* system based on the proposed sensor (GCE/CuO NPs) is presented in Scheme 2.

**Fig. 4:**
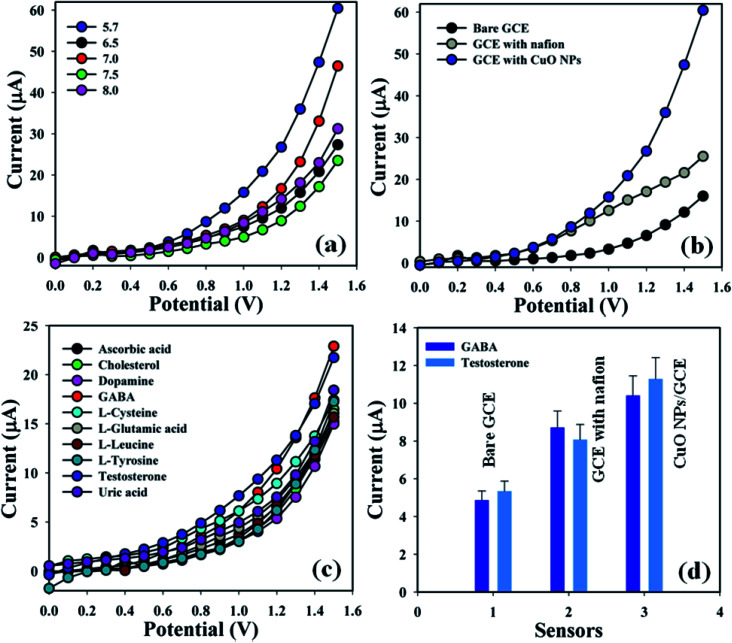
(a) pH optimization, (b) bare and coated electrode, (c) selectivity study, and (d) bar diagram presentation of control experiment at +1.2 V with error limit 10.0%.

Accordingly, the fabricated electrodes were examined in buffer phases (pH = 5.7–8.0) in order to find out the appropriate phosphate buffer phase for conducting the whole sensing experiment. An elevated electrical response was found at pH = 5.7 compared with additional modified electrodes (Fig. 4a). Augmented electrical responses in the working electrode were found based on the GCE covered with CuO NPs, compared with bare GCE and GCE with nafion (Fig. 4b). A selectivity study was performed in the buffer phase (pH = 5.7, amount = 10.0 mL, and conc. = 100.0 mM) in the presence of different biological molecules – GABA, ascorbic acid, cholesterol, dopamine, l-glutamic acid, l-glutamic acid, l-luciene, l-tyrosine, testosterone, and uric acid (amount ≈ 25.0 μL and concentration = 1.0 μM) – using a variety of modified electrodes, combined with the proposed sensor (GCE/CuO NPs). It was principally observed that the sensor was more sensitive towards γ-amino-butyric acid and testosterone compared with the other biomolecules (Fig. 4c). A control experiment was conducted in the absence and presence of GABA and TST (concentration = 1.0 μM, amount ≈ 25.0 μL) in buffer phase (pH = 5.7, amount = 10.0 mL, and conc. = 100.0 mM) to find out the electrical response towards the proposed sensor (GCE/CuO NPs). The designed sensor showed higher response towards GABA and TST compared with other sensors such as bare GCE and GCE with nafion (Fig. S1†). Fig. 4d is the bar diagram presentation of the control experiment at +1.2 V with error limit 10.0%.

**Fig. 5:**
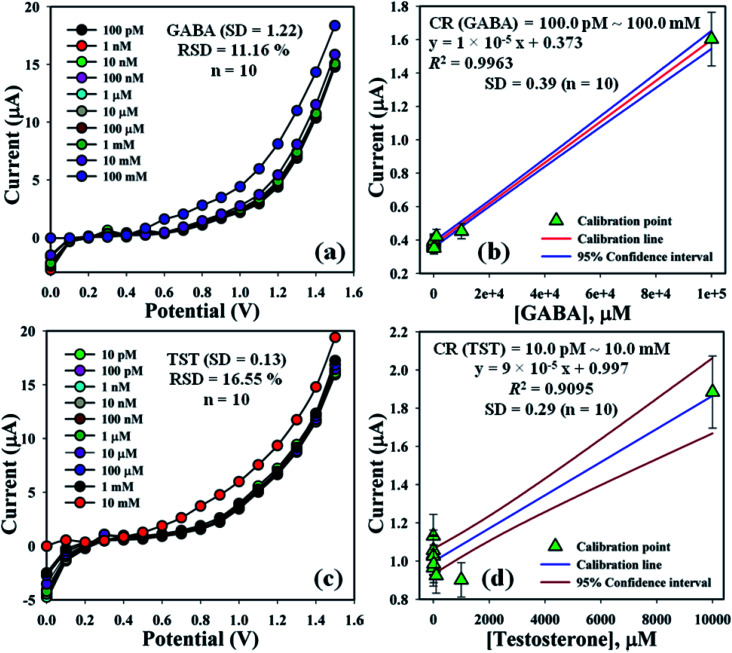
Concentration variation study and calibration diagram with error limit 10.0%: (a and b) GABA and (c and d) testosterone.

The electrochemical signals of GABA and TST with the proposed sensor (GCE/CuO NPs) were examined to find the change in current response. This was based on the eventual function of biological molecule detection in a concentration range, GABA: 100.0 pM to 100.0 mM and TST: 10.0 pM to 10.0 mM. It was recorded that electrochemical responses increased from lower to higher concentrations of GABA and TST {GABA (SD = 1.22, RSD = 11.16%, and *n* = 10) and TST (SD = 0.13, RSD = 16.55%, and *n* = 10)} at +1.4 V (Fig. 5a–c). Calibration curves were plotted from GABA and TST concentrations at +0.6 V and found a linear response {(GABA: *R*^2^ = 0.9963, *y* = 1 × 10^−5^*x* + 0.373, SD = 0.39, and *n* = 10) and (TST: *R*^2^ = 0.9095, *y* = 9 × 10^−5^*x* + 0.997, SD = 0.29, and *n* = 10)} with an error limit 10.0%. Analytical parameters of the proposed sensor such as sensitivity were found as GABA (316.46 pA μM^−1^ cm^−2^) and TST (2848.10 pA μM^−1^ cm^−2^). Due to the concentration range of GABA (100.0 pM to 100.0 mM) and TST (10.0 pM to 10.0 mM), LOD were expressed to 11.70 pM and 96.67 pM, respectively. The LOQ was found for GABA (39.0 pM) and TST (322.2 pM) from calibration curves based on eqn (x)–(xii).^19–23^ Where, *m* = slope of the calibration bend (1 × 10^−5^ and 9 × 10^−5^), *A* = dynamic surface area of GCE (diameter = 0.0316 cm^2^), SD = standard deviation (0.39 and 0.29) of GABA and TST concentration, calibrated at +0.6 V (Fig. 5b–d).x
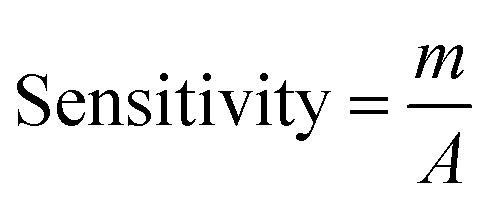
xi
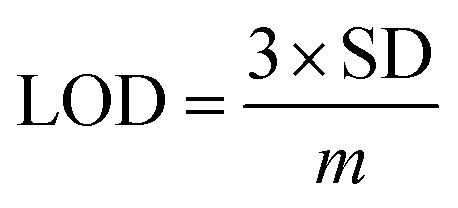
xii
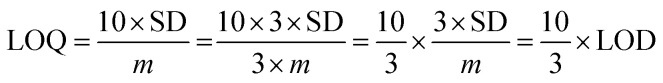


**Fig. 6:**
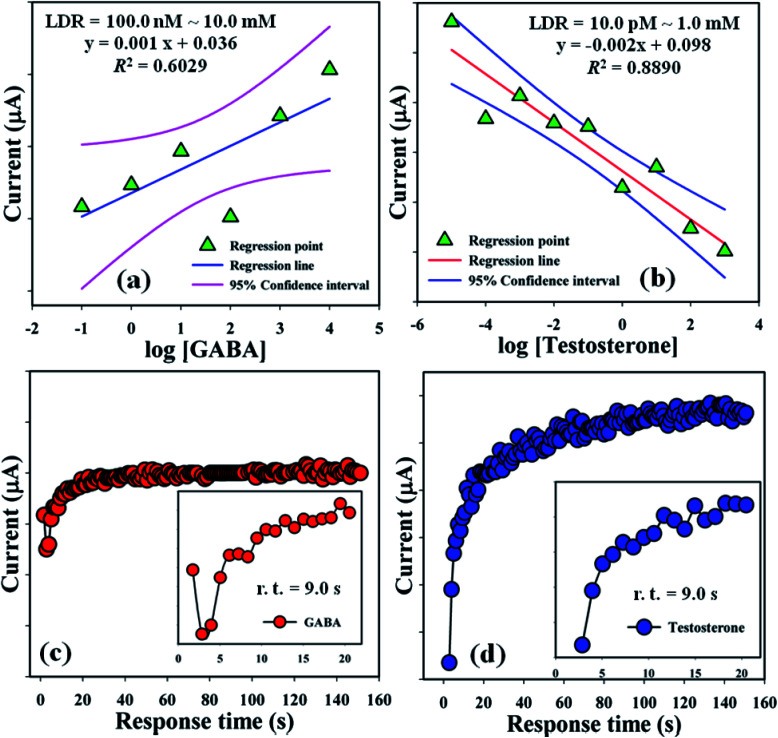
LDR plot and response time: (a and c) GABA and (b and d) testosterone.

Linear dynamic ranges (100.0 nM to 10.0 mM and 10.0 pM to 1.0 mM) were achieved from the calibration curves and found to be linear (*R*^2^ = 0.6029 and 0.8890) with linear regression equation, *y* = 0.001*x* + 0.036 and *y* = 0.002*x* + 0.098, respectively, for GABA and TST (Fig. 6a and b). Response time of GABA and TST (amount = 25.0 μL and conc. = 1.0 μM) with the proposed sensor (GCE/CuO NPs) was examined in buffer phase (pH = 5.7, amount = 10.0 mL, and conc. = 100.0 mM) and found to be 9.0 s and 9.0 s sequentially (Fig. 6c and d). A comparison of biological molecules (GABA and testosterone) detection using different modified sensors with various methods is presented in Table 1.

**Table 1:** Detection of GABA and testosterone using different modified electrochemical sensors^a^

BM	Sensors	pH	Sensitivity	LOD (pM)	LOQ (pM)	LDR	r.t. (s)	RP (%)	RA (%)	Ref.
GABA	AgNPs	3.8	—	57.7 mg L^−1^	79.2 mg L^−1^	—	—	—	—	2
	Fe_3_O_4_@SiO_2_@meso SiO_2_ microspheres	—	0.94 nm/log *M*		3.51 × 10^−13^ M	—	—	—	—	6
	GCE/CuO NPs	5.7	316.46 pA μM^−1^ cm^−2^	11.70	39.0	100.0–10.0 (nM to mM)	9.0	55.0	89.0	This work
TST	CuO–CeO_2_ NSs/GCE	—	27.36 μA μM^−1^ cm^−2^	9.30 pM	—	0.01–0.01 (nM to mM)	25	—	—	24
	MIPs materials	—	—	0.5 nM	—	0.5–20.0 (nM to nM)	—	—	—	25
	GO materials	—	—	0.4 fM	—	1.0–1.0 (fM to μM)	—	—	—	26
	CTAB/GC	—	—	1.18 nM	—	10.0–70.0 nM	—	—	—	27
	GCE/CuO NPs		2848.10 pA μM^−1^ cm^−2^	96.67	322.2	10.0–1.0 (pM to mM)	9.0	61.0	98.0	This work

a BM: biological molecules, l-LA: l-lactic acid, UA: uric acid, GSH: glutathione, LOD: limit of detection, LOQ: limit of quantification, LDR: linear dynamic range, r.t.: response time, RP: reproducibility, and RA: repeatability.

#### 3.4.2 Examination of sensor performances

**Fig. 7:**
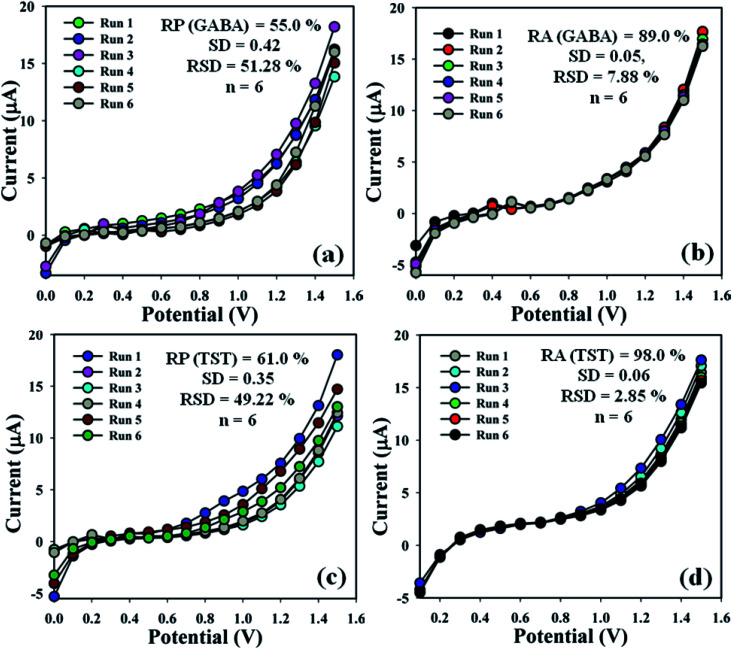
Reproducible and repeatability studies of the fabricated GCE/CuO NPs sensor: (a and b) GABA and (c and d) testosterone.

The efficiency of the proposed sensor (GCE/CuO NPs) was examined in different days to find out the electrochemical effectiveness, such as reproducibility and repeatability. A sequence of six consecutive measures of GABA and TST (amount = 25.0 μL and conc. = 1.0 μM) were conducted by means of various modified electrodes in an identical system: phosphate buffer phase (pH = 5.7, amount = 10.0 mL, and conc. = 100.0 mM). As a result, a good reproducible (RP) response of the sensor probe in the calibrated potential (+0.6 V) was found {(GABA = 55.0%, SD = 0.42, RSD = 51.28%, and *n* = 6) and (TST = 61.0%, SD = 0.35, RSD = 49.22%, and *n* = 6)}, (Fig. 7a–c and Table S1†). Electrochemical responses of the proposed sensor (GCE/CuO NPs) were analyzed for the purpose of considering long storage time. An enhancement of storage capability of the proposed sensor was considered compared to similar electrodes for GABA and TST at concentrations of 1.0 μM and amount 25.0 μL at buffer phase (pH = 5.7, amount = 10.0 mL, and conc. = 100.0 mM). Repeatability (RA) was found at the calibrated potential of +0.6 V as {(GABA = 89.0%, SD = 0.05, RSD = 7.88%, and *n* = 6) and (TST = 98.0%, SD = 0.06, RSD = 2.88%, and *n* = 6)}, (Fig. 7b–d and Table S1†). It was notably found that the proposed sensor (GCE/CuO NPs) can be used repeatedly without significant loss of analyzing capability.^28–33^

#### 3.4.3 Examination of interference effects

**Fig. 8:**
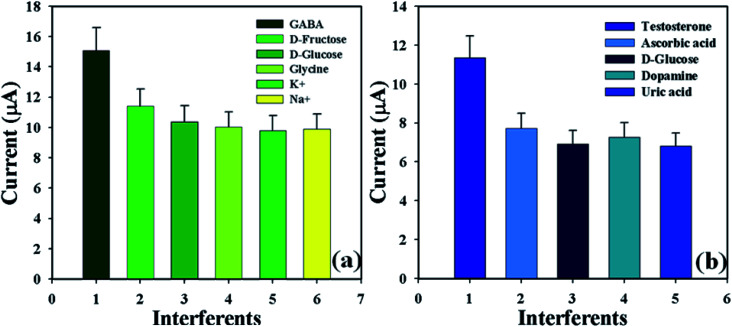
Bar diagram presentation of interference effects at +1.2 V with error limit 10.0%: (a) GABA and (b) testosterone.

Analysis of interference effects is a good investigative experiment to consider non-target biological molecules that may have similar activities towards the proposed sensor.^34,35^ Interfering agents such as d-fructose, d-glucose, glycine, K^+^, and Na^+^ for GABA; and ascorbic acid, d-glucose, dopamine, and uric acid for TST, were examined separately in order to find out the electrochemical responses towards the proposed sensor (GCE/CuO NPs). These analyses were performed in phosphate buffer phase (pH = 5.7, amount = 10.0 mL, and conc. = 100.0 mM) using the fabricated electrode for GABA and TST (amount = 25.0 μL and conc. = 1.0 μM) and interfering agents (amount = 25.0 μL and conc. = 10.0 μM). The efficiency of interfering agents, GABA, and TST towards the anticipated sensor were calculated at the calibrated potential (+0.6 V). It is highlighted here that the efficiency of GABA and TST was found to be 100.0% (Fig. S2 and Table S2†). Fig. 8 shows the bar diagram appearance of interference efficiency at +1.2 V with error limit 10.0%. It was found that the GCE/CuO NPs sensor did not show any significant electrochemical response towards the interfering agents that were at ten times higher concentration than GABA and TST. The planned sensor (GCE/CuO NPs) can be a good analytical application for the detection of the selected biological molecules with good sensitivity.

#### 3.4.4 Real samples analysis

**Fig. 9:**
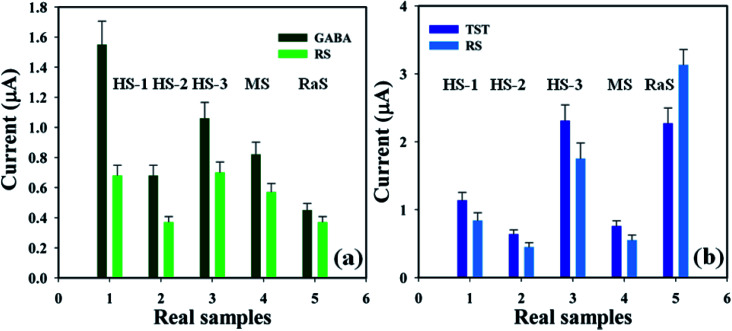
Real sample analysis with error limits 10.0%: (a) GABA and (b) testosterone.

A detailed analysis was conducted in order to examine real biological samples – human serum (HS), mouse serum (MS), and rabbit serum (RaS) – using the proposed sensor (GCE/CuO NPs) in order to identify GABA and TST concentrations based on a standard addition technique.^36,37^ A fixed quantity (∼25.0 μL) of each real sample was examined in phosphate buffer phase (pH = 5.7, amount = 10.0 mL, and conc. = 100.0 mM). The concentration of GABA and TST was calculated at the calibrated potential (+0.6 V) in HS, MS, and RaS, which showed that the current–voltage process can be a good experimental tool for the analysis of biological molecules (Fig. 9 and Table 2).

**Table 2:** Real samples analysis using GCE/CuO NPs sensor by electrochemical method^a^

BM	ME	CA (μM, 25.0 μL)	OC (μA)	RSA (25.0 μL)	ROC (RSA, μA)				FC (μM)	*R* (%)	SD (*n* = 3)	RSD% (*n* = 3)
					R1	R2	R3	A				
GABA	1	1.0	1.55	HS-1	0.67	0.75	0.63	0.68	0.44	44	0.06	8.94
	2	1.0	0.68	HS-2	0.39	0.36	0.36	0.37	0.54	54	0.02	4.68
	3	1.0	1.06	HS-3	0.71	0.71	0.68	0.70	0.66	66	0.02	2.47
	4	1.0	0.82	MS	0.61	0.54	0.55	0.57	0.70	70	0.04	6.68
	5	1.0	0.45	RaS	0.36	0.42	0.34	0.37	0.82	82	0.04	11.15
TST	1	1.0	1.14	HS-1	0.90	0.81	0.81	0.84	0.74	74	0.05	6.19
	2	1.0	0.64	HS-2	0.44	0.44	0.46	0.45	0.70	70	0.01	2.59
	3	1.0	2.31	HS-3	1.55	1.82	1.87	1.75	0.76	76	0.17	9.86
	4	1.0	0.76	MS	0.55	0.55	0.56	0.55	0.72	72	0.006	1.04
	5	1.0	2.27	RaS	3.42	2.99	2.97	3.13	1.38	138	0.25	8.13

a BM: biomolecules, ME: modified electrode, CA: concentration added, GABA: γ-amino-butyric acid, TST: testosterone, OC: observed current, RSA: real sample added, ROC: respective observed current, RSA: real sample added, R: reading, A: average, FC: found concentration, *R*: recovery, SD: standard deviation, RSD: relative standard deviation, HS: human serum, MS: mouse serum, and RaS: rabbit serum.

## 4. Conclusion

A wet-chemical technique was applied to synthesise low-dimensional CuO NPs which were then modified on a GCE to produce a GCE/CuO NPs sensor. This sensor was used to detect biological molecules GABA and TST, using a reliable current–voltage technique with good analytical performance. Good analytical performance of the proposed sensors were achieved from the perspective of methodological key parameters such as sensitivity, LOD, LOQ, LDR, response time, reproducibility, and repeatability. A significant approach can be inferred from our development, regarding the identification of biological molecules in real samples with CuO nanoparticles fabricated with GCE using a conducting polymer matrix. Overall, we introduced a promising enzyme-free approach using a metal oxide nanomaterial electrochemical technique for the detection of biochemicals for safe use in healthcare and biomedical fields.

## Author contributions

MMH planned and accomplished the experiment. MMH also wrote and corrected the whole manuscript. MMR has performed the SEM, XPS, and XRD to analyze the CuO NPs. JU has performed morphological analysis. AMA and MMR corrected the manuscript. All authors approved the corrected manuscript to publish in this journal in the current state.

## Conflicts of interest

The authors declare that there is no conflict of interest in this research article.

## Supplementary Material

RA-011-D1RA02709C-s001
