# Supplementary material for: An enzyme free simultaneous detection of γ-amino-butyric acid and testosterone based on copper oxide nanoparticles
Source: RSC Adv. 2021 Jun 11;11(34):20794–805. doi: 10.1039/d1ra02709c (PMC9033999; doi:10.1039/d1ra02709c)
Supplement: RA-011-D1RA02709C-s001 [file RA-011-D1RA02709C-s001.pdf]

Electronic Supplementary Material (ESI) for RSC Advances.
This journal is © The Royal Society of Chemistry 2021

An enzyme free simultaneous detection of γ-amino-butyric acid and testosterone based on copper oxide nanoparticles

Mohammad Musarraf Hussain[1,2,3*], Abdullah M. Asiri[1, 2], Jamal Uddin[4], Mohammed M. Rahman[1, 2*]

[1]Department of Chemistry, Faculty of Science, King Abdulaziz University, Jeddah 21589, P.O. Box 80203, Saudi Arabia

[2]Center of Excellence for Advanced Materials Research (CEAMR), King Abdulaziz University, Jeddah 21589, P.O. Box 80203, Saudi Arabia

[3]Department of Pharmacy, Faculty of Life and Earth Sciences, Jagannath University, Dhaka-1100, Bangladesh

[4]Center for Nanotechnology, Department of Natural Sciences, Coppin State University, Baltimore, MD 21216, USA

Electronic supplementary materials (ESM)

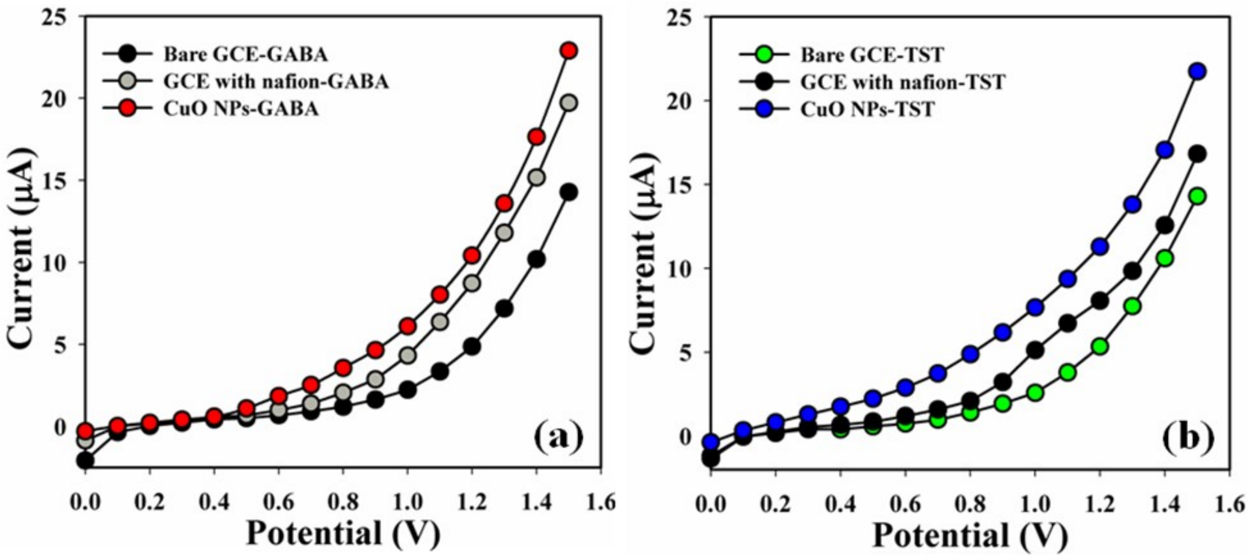

Fig. S1 Control experiment of (a) GABA and (b) Testosterone

Table S1 Evaluation of CuO NPs sensor performance (GABA and Testosterone)

BM	Replicates	Current (μA)	Reproducibility (%)		Current (μA)	Repeatability (%)	
			Individual	Average		Individual	Average
GABA	1	1.49	100	55	0.68	100	89
	2	1.11	75		0.63	93	
	3	0.80	54		0.59	87	
	4	0.56	38		0.60	88	
	5	0.32	22		0.59	87	
	6	0.64	43		0.54	79	
Testosterone	1	1.18	100	61	2.09	100	98
	2	0.50	42		1.98	95	
	3	0.39	33		1.97	94	
	4	0.55	47		2.09	100	
	5	1.15	98		2.09	100	
	6	0.52	44		2.07	99	

Reproducibility and repeatability of GABA and Testosterone of replicate 1 has been considered to be 100 %

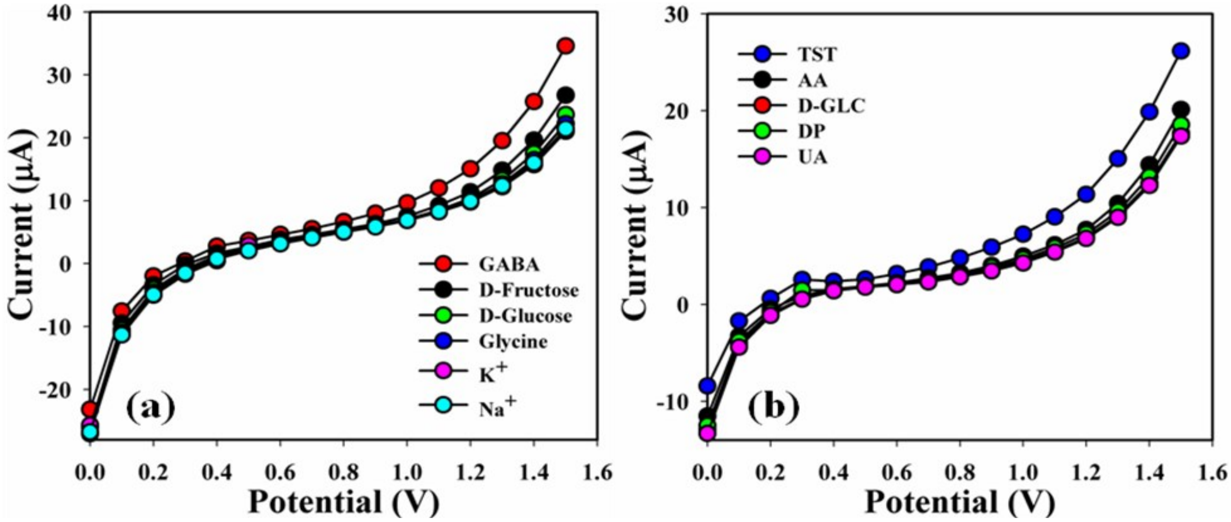

Fig. S2 Interference effect examination (a) GABA and (b) TST

Table S2 Interference effect examination towards GCE/CuO NPs sensor

BM	IA	Observed current (μA)				IE (%)	SD (n = 3)	RSD (%) (n = 3)
		**R1**	**R2**	**R3**	**Average**			
GABA	GABA	5.42	4.41	4.08	4.64	100	0.70	15.06
	D-FRCT	4.10	3.71	3.63	3.81	82	0.25	6.59
	D-GLC	3.64	3.41	3.47	3.51	76	0.12	3.40
	Glycine	3.54	3.15	3.06	3.25	70	0.26	7.85
	K^+	3.37	2.90	3.43	3.23	70	0.29	8.98
	Na^+	3.49	2.97	2.98	3.15	68	0.30	9.45
Testosterone	TST	4.22	2.81	2.45	3.16	100	0.94	29.60
	AA	2.53	2.12	2.07	2.24	71	0.25	11.27
	D-GLC	2.15	1.85	2.10	2.03	64	0.16	7.90
	DP	2.37	2.04	2.08	2.16	68	0.18	8.32
	UA	2.10	2.02	2.01	2.04	65	0.05	2.41
